# A Comparison of Subcutaneous with Intravenous and Intrathecal Administration of Tetanus Antitoxin in Experimental Tetanus

**Published:** 1918-01

**Authors:** 


***A Comparison of Subcutaneous with Intravenous and Intrathecal Administration of Tetanus Antitoxin in Experimental Tetanus.*** By F. Golla, Capt. R. A. M. C. (T. F.). Abs. from the *Lancet*, May 5, 1917.

For the purpose of the comparison announced in the title, G. performed a series of experiments on rabbits and cats, because the higher resistance in these animals to tetanus makes the picture of tetanus in them comparable to that seen in man after a preventive treatment by serum.

4 rabbits were given equal injections of toxin. 24 hours later each rabbit was given 75 U.S.A. units of antitoxin — 2 by the intrathecal route and 2 by the subcutaneous. The 2 treated intrathecally recovered completely after three days. The 2 treated subcutaneously developed much more serious tetanus : one of them died on the 4th day, and in the other, which finally recovered, rigidity lasted for a month.

3 cats were given equal doses of toxin. 2 of them received 75 U.S.A. units of antitoxin 24 hours later, one intrathecally and the other subcutaneously. The third cat was given no injection of serum. The cat that received an intrathecal injection recovered completely in three days. The one that was treated subcutaneously developed marked rigidity of the leg which lasted until it was killed a week later. The cat that received no treatment developed a case similar to the latter, except that its anterior left extremity was also affected.

10 rabbits with equal doses of toxin were divided into groups, some being treated with antitoxin at the end of 24 hours before symptoms had appeared, some at the end of 36 hours when slight local spasms were observed, and the last group were treated at the end of 48 hours when the local spasms and weakness of the extremities were more pronounced. In all instances, those treated by the intrathecal route showed the best progress. In the group treated before the symptoms had appeared, the animal receiving intrathecal injections recovered after 4 days. The animal treated subcutaneously, however, died on the 4th day. In the groups treated after symptoms appeared, all of the 4 animals treated subcutaneously died within 4 days. 1 of those treated intrathecally recovered, the other 3 died in from 5 to 6 days.

12 rabbits, in the last experiment recorded, were divided into 3 equal groups. One of these was treated intrathecally, another intravenously, and the third subcutaneously. Two in each group were treated after 24 hours and two after 48 hours. All of the animals treated subcutaneously died on the 3rd day. All but one of the remaining 8, treated intravenously or intrathecally, recovered. The one which died had been treated intrathecally.

The author concludes from this last experiment that the intrathecal method, even when employed 24 hours later than the subcutaneous route, gives vastly superior results.

